# Acute restraint stress reverses impaired LTP in the hippocampal CA1 region in mouse models of Alzheimer’s disease

**DOI:** 10.1038/s41598-019-47452-6

**Published:** 2019-07-29

**Authors:** Ming Wang, Vijay Sankar Ramasamy, Manikandan Samidurai, Jihoon Jo

**Affiliations:** 1 NeuroMedical Convergence Lab, Biomedical Research Institute, Chonnam National University Hospital, Jebong-ro, Gwangju, 501-757 Republic of Korea; 2 Department of Biomedical Sciences, BK21 PLUS Center for Creative Biomedical Scientists at Chonnam National University, Research Institute of Medical Sciences, Chonnam National University Medical School, Gwangju, 501-757 South Korea; 3 Department of Neurology, Chonnam National University Medical School, Gwangju, 501-757 Republic of Korea

**Keywords:** Molecular neuroscience, Alzheimer's disease, Cellular neuroscience

## Abstract

Acute stress facilitates long-term potentiation (LTP) in the mouse hippocampus by modulating glucocorticoid receptors and ion channels. Here, we analysed whether this occurs in mouse models of Alzheimer’s disease (AD) with impaired LTP induction. We found that a brief 30 min restraint stress protocol reversed the impaired LTP assessed with field excitatory postsynaptic potential recordings at cornu ammonis 3-1 (CA3-CA1) synapses in both Tg2576 and 5XFAD mice. This effect was accompanied by increased phosphorylation and surface expression of glutamate A1 (GluA1) -containing α-amino-3-hydroxy-5-methyl-4-isoxazolepropionic acid receptors (AMPARs). Moreover, enhanced LTP induction and GluA1 phosphorylation were sustained up to 4 h after the stress. Treatment with 200 nM dexamethasone produced similar effects in the hippocampi of these mice, which supports the glucocorticoid receptor-mediated mechanism in these models. Collectively, our results demonstrated an alleviation of impaired LTP and synaptic plasticity in the hippocampal CA1 region following acute stress in the AD mouse models.

## Introduction

Alzheimer’s disease (AD) is the most common form of dementia in the aging population, and sporadic late-onset AD accounts for most cases of AD^1^. The major hallmarks of the disease are progressive memory loss and the accumulation of amyloid-β (Aβ) in the brain^2,3^. Aβ disrupts synaptic plasticity and impairs memory by modulating the mechanisms controlling long-term potentiation (LTP)^4,5^.

LTP is the long-lasting increase in synaptic strength that can be induced by tetanic stimulation of afferent fibres^6,7^. It is believed to reflect long-term changes in the synaptic efficacy. Thus hippocampal LTP is regarded as a cellular model of learning and memory^6^. LTP is triggered by high-frequency stimulation (HFS) in the cornu ammonis1 (CA1) area of the hippocampus, and requires postsynaptic activation of *N*-methyl-d-aspartate receptors (NMDARs) as well as surface insertion of α-amino-3-hydroxy-5-methyl-4-isoxazolepropionic acid receptors (AMPARs)^8^, the trafficking of which underlies excitatory synaptic plasticity^9^. The LTP-associated increases in AMPAR surface expression and synaptic strength have been attributed to the influx of Ca^2+^ through NMDARs and subsequent activation of protein kinase A and calcium/calmodulin-dependent protein kinase II (CaMKII). However, exogenously applied Aβ or amyloid precursor protein (APP) overexpression in AD mouse models induces the removal of AMPARs from synapses, resulting in impaired hippocampal LTP^10–14^. Aβ impairs AMPAR trafficking and function by reducing the activation^15^ and synaptic distribution^16^ of CaMKII and by calcineurin- and protein phosphatase-1-mediated dephosphorylation of AMPAR subunits^17^. Aβ may also affect hippocampal LTP by either inducing NMDAR-mediated excitotoxicity^18^ or reducing NMDAR surface expression via endocytosis.

Impaired LTP and cognition in mouse models of AD is exacerbated by chronic stress^19^. Chronic stress also impairs hippocampal LTP in the dentate gyrus and CA1 in rats^20,21^, which is associated with reduced CaMKII levels^22^ and a selective decrease in AMPAR-mediated synaptic excitation^23^. The suppressed AMPAR and NMDAR expression and associated synaptic transmission lead to cognitive impairment^24^. By contrast, short or acute stress, such as the forced swim test and elevated plus maze task for 20 min, facilitates NMDAR and AMPAR expression and glutamatergic transmission in rat prefrontal cortex, a brain region mainly involved in working memory^25^. Furthermore, acute stress exposure increases AMPAR surface expression and enhances LTP induction in the CA1 region of rat hippocampus^26^.

The present study was conducted to determine whether acute stress can reverse impaired hippocampal LTP in mouse models of AD. We used restraint as a paradigm to induce acute stress^27^ in well-characterised transgenic mouse models, Tg2576 and 5XFAD, which express genes associated with familial AD and exhibit impaired LTP^28,29^. To characterise LTP and synaptic plasticity in the hippocampi of these mice, we recorded field excitatory postsynaptic potentials (fEPSPs) in the CA1, and measured the phosphorylation and surface expression of GluA1 subunits of AMPARs. The effects of stress were validated *in vivo* and *ex vivo* in experiments using the glucocorticoid receptor agonist dexamethasone.

## Results

### Acute stress rescues impaired LTP in the hippocampal CA1 region of AD mice

**Figure 1:**
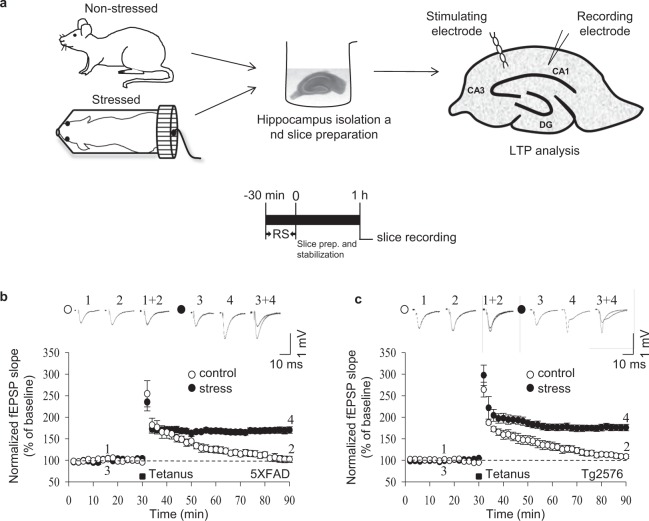
Acute stress rescues LTP impairment in AD mice. (**a**) Schematic diagram of the restraint stress protocol and field recording on a hippocampal slice. After 30 min of baseline recordings, LTP was induced in CA1 neurons with tetanic stimulation (two trains of 100 Hz, 100 pulses; black square). Exposure to 30 min of restraint stress rescued impaired LTP in 5XFAD (**b**) and Tg2576 (**c**) mice. Error bars represent the SEMs (*n* = 6/group).

In the first set of experiments, we determined whether acute stress alters LTP in the CA1 region by recording fEPSPs in acutely prepared hippocampal slices from either 5XFAD or Tg2576 mice (Fig. 1a). The baseline recordings were stable for 30 min, with no significant difference between stressed and control unstressed mice. However, further recordings for 60 min following HFS revealed that LTP was not induced in slices from control 5XFAD (*n* = 6 [six slices from six animals per group]) or control Tg2576 (*n* = 6) mice (Fig. 1b,c, respectively). By contrast, LTP was induced by HFS in slices from stressed 5XFAD [t = −6.855, df = 9; *p* < 0.01, *n* = 6, unpaired *t* test; Fig. 1b] and stressed Tg2576 [t = −11.135, df = 6; *p* < 0.01, *n* = 6, unpaired *t* test; Fig. 1c] mice. Additional experiments in slices from wild-type mice confirmed that LTP is induced by HFS in slices from unstressed control mice (*n* = 5) and that acute stress significantly enhances the magnitude of the LTP [t = −8.8, df = 9; *p* < 0.01, *n* = 5–6, unpaired *t* test; Supplementary Fig. 1] (Table 1). Altogether, these data confirm that acute stress potentiates LTP and suggest that acute stress can rescue impaired LTP in mouse models of AD.

**Table 1:** Effect of acute stress on LTP, Surface expression and phosphorylation of AMPA - GluA1.

	fEPSP slope		sGluA1		pS845-GluA1	
	C	S	C	S	C	S
5XFAD	102 ± 7%	170 ± 8%	100%	215 ± 8%	100%	253 ± 5%
Tg2576	109 ± 4%	176 ± 7%	100%	294 ± 9%	100%	236 ± 5%
Wild type	148 ± 4%	203 ± 5%	100%	176 ± 25%	100%	232 ± 43%

### Acute stress increases surface expression and GluA1 phosphorylation

**Figure 2:**
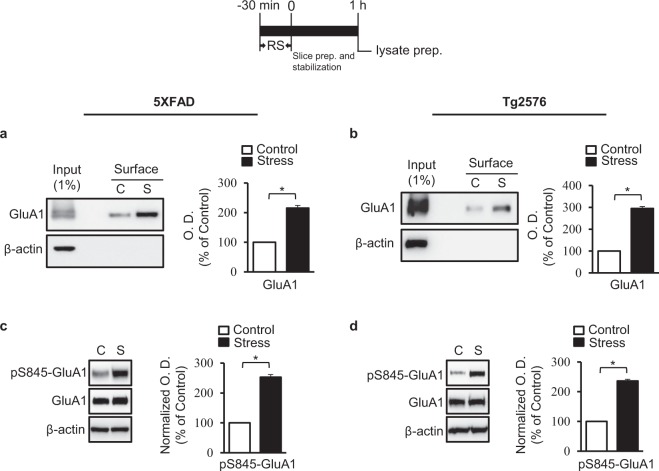
Acute stress induces phosphorylation and recruitment of GluA1 receptors to the cell surface. Representative immunoblots and densitometry analysis showing GluA1 surface expression in hippocampi of 5XFAD mice (*n* = 4/group) (**a**) and Tg2576 mice (*n* = 3/group) (**b**). Representative immunoblots and densitometry analysis showing the amount of pS845-GluA1 and total GluA1 in hippocampi from 5XFAD mice (*n* = 5/group) (**c**) and Tg2576 mice (*n* = 4/group) (**d**). C, unstressed control; S, stressed. Error bars indicate SEMs; *P < 0.01. Full length blots are presented in Supplementary Fig. 3.

We next performed biotinylation assays to evaluate the surface expression of AMPAR GluA1 subunits in hippocampal tissues from control and acutely stressed AD model mice. Western blots revealed that the surface expression of GluA1 was higher in stressed mice than in controls for the 5XFAD model [t = −6.469, df = 9; *p* < 0.01, *n* = 4, unpaired *t* test; Figs 2a, S3] and the Tg2576 model [t = −6.841, df = 4; *p* < 0.01, *n* = 3, unpaired *t* test; Figs 2b, S3]. Similar results were obtained with wild-type mice exposed to acute stress [t = −5.2, df = 4; *p* < 0.05, *n* = 3, unpaired *t* test; Figs S2a, S6].

The increase in surface expression was accompanied by an increase in phosphorylation at serine 845 (pS845). Western blotting analyses showed that exposure to acute stress increased the levels of pS845-GluA1 in hippocampi from 5XFAD mice [t = −3.730, df = 8; *p* < 0.01, *n* = 5, unpaired *t* test; Figs 2c, S3] and Tg2576 mice [t = −4.988, df = 6; *p* < 0.01, *n* = 4, unpaired *t* test; Figs 2d, S3]. The acute stress-induced phosphorylation also occurred in wild-type mice [t = −17.5, df = 4; *p* < 0.01, *n* = 4, unpaired *t* test; Figs S2b, S6]. The total levels of GluA1 in the hippocampus did not differ between stressed and control animals in both models (Fig. 2c,d).

### Sustained hippocampal LTP and GluA1 phosphorylation

**Figure 3:**
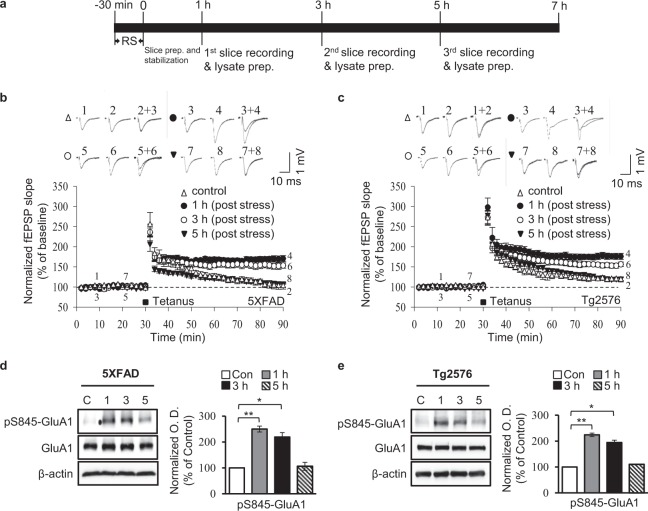
Acute stress rescues LTP and induces pS845-GluA1 expression in a time-dependent manner. (**a**) Schematic representation of the restraint stress experimental setup. Representative LTP magnitudes at 1, 3 and 5 h after stress; LTP induced by acute stress was maintained for >3 h in 5XFAD (**b**) and Tg2576 (**c**) mice (*n* = 5–6/group). Representative immunoblots and densitometry analysis showing the amount of pS845-GluA1 and total GluA1 in hippocampi from 5XFAD mice (*n = *5/group) (**d**) and Tg2576 mice (*n* = 6/group) (**e**). C, unstressed control; 1, 3 and 5 h post-stress. Error bars indicate SEMs; *P < 0.05, **P < 0.01. Full length blots are presented in Supplementary Fig. 4.

To determine the time course of enhanced LTP and hippocampal GluA1 phosphorylation, we analysed fEPSPs 1, 3 and 5 h after the restraint stress (Fig. 3a). The magnitude of LTP in hippocampal slices prepared from stressed 5XFAD mice remained higher after 1 and 3 h [1 h: F(3,17) = 32.6; *p* < 0.01 and 3 h: *p* < 0.01 respectively, n = 5–6, one-way analysis of variance [ANOVA] with Scheffe test; Fig. 3b], but was not significantly different from controls after 5 h [5 h: F(3,17) = 32.6; *p* = 0.882, *n* = 6, ANOVA with Scheffe test; Fig. 3b]. Similarly, Tg2576 mice exhibited higher LTP magnitudes at 1 and 3 h after acute stress [1 h: F(3,18) = 24.7; *p* < 0.01 and 3 h: *p* < 0.01 respectively, n = 5–6, ANOVA with Scheffe test; Fig. 3c], with fEPSP magnitudes that did not differ from control slices at 5 h [5 h: F(3,18) = 24.7; *p* = 0.992, *n* = 6, ANOVA with Scheffe test; Fig. 3c].

Western blot analyses of hippocampal samples taken from 5XFAD mice indicated that the levels of pS845-GluA1 were elevated 1 and 3 h after the restraint stress [1 h: F(3,11) = 13.4; *p* < 0.01 and 3 h: *p* < 0.05 respectively, n = 5, ANOVA with Games-Howell test; Figs 3d, S4] and returned to control levels by 5 h [5 h: F(3,11) = 13.4; *p* = 1.0, *n* = 6, ANOVA with Games-Howell test; Figs 3d, S4]. Similarly, pS845-GluA1 levels from Tg2576 mice were increased at 1 and 3 h after the restraint stress [1 h: F(3,7) = 7.6; *p* < 0.01 and 3 h: *p* < 0.05 respectively, n = 6, ANOVA with LSD test; Figs 3e, S4], with control levels observed at 5 h [5 h: F(3,7) = 7.6; *p* = 0.946, *n* = 6, ANOVA with LSD test; Figs 3e, S4]. Collectively, these results indicate that the acute stress-enhanced LTP induction and GluA1 phosphorylation persisted for more than 3 h in two mouse models of AD.

### Glucocorticoid receptor agonist mimics the acute stress effects in AD mouse models

**Figure 4:**
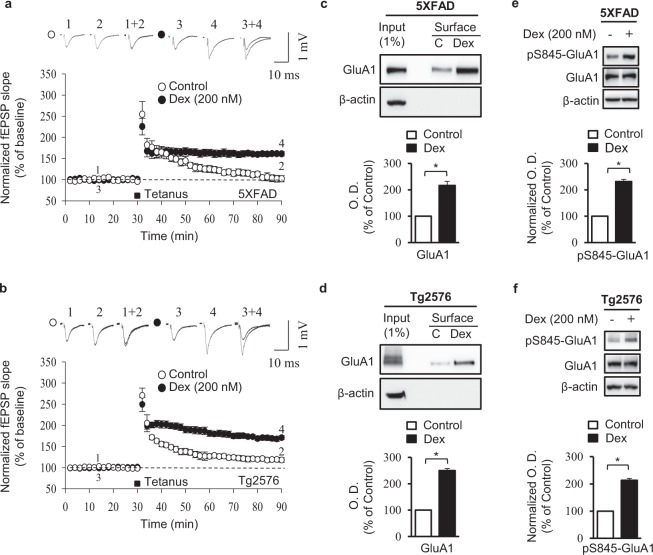
Glucocorticoid receptors regulate acute stress-mediated LTP and pS845-GluA1 expression in AD mice Representative LTP analyses in hippocampal slices treated with dexamethasone (Dex [200 nM]) from 5XFAD (*n* = 6/group) (**a**) and Tg2576 (*n* = 5/group) mice (**b**). Representative immunoblots and densitometry analyses showing GluA1 surface expression in hippocampi from 5XFAD (*n* = 6/group) (**c**) and Tg2576 (*n* = 5/group) (**d**) mice. Representative immunoblots and densitometry analyses showing pS845-GluA1 and total GluA1 expression in hippocampi from 5XFAD (*n* = 5/group) (**e**) and Tg2576 (*n* = 4/group) mice (**f**). C, untreated control. Error bars indicate SEMs; *P < 0.01. Full length blots are presented in Supplementary Fig. 5.

We next evaluated whether a glucocorticoid receptor agonist would induce effects similar to acute restraint stress on hippocampal LTP in 5XFAD and Tg2576 mice. Acutely prepared hippocampal slices were perfused with 200 nM dexamethasone (a dose that enhances LTP in mice^26^) while fEPSP recordings were acquired. Baseline levels were similar between treated and untreated slices. fEPSP slopes following HFS were significantly higher in slices treated with 200 nM dexamethasone than in untreated slices from 5XFAD mice [t = −7.1, df = 9; *p* < 0.01, *n* = 6, unpaired *t* test; Fig. 4a] and Tg2576 mice [t = −7.2, df = 8; *p* < 0.01, *n* = 6, unpaired *t* test; Fig. 4b], demonstrating enhanced LTP in the presence of the glucocorticoid agonist.

Results from the surface biotinylation assays revealed that 30 min dexamethasone treatment significantly increased the surface levels of GluA1 in hippocampal tissues from 5XFAD [t = −4.4, df = 6; *p* < 0.01, *n* = 6, unpaired *t* test; Figs 4c, S5] and Tg2576 [t = −29.3, df = 4; *p* < 0.01, *n* = 5, unpaired *t* test; Figs 4d, S5]. Accordingly, the levels of pS845-GluA1 in 5XFAD [t = −9.4, df = 4; *p* < 0.01, *n* = 5, unpaired *t* test; Figs 4e, S5] and Tg2576 [t = −4.2, df = 4; *p* < 0.01, *n* = 4, unpaired *t* test; Figs 4f, S5] mice were significantly increased by dexamethasone treatment. Dexamethasone treatment did not affect total GluA1 levels (Figs 4e,f, S5). These results support the previous observations on the effects of acute stress and glucocorticoids on hippocampal LTP enhancement in mice.

## Discussion

The results of the present study show that exposure to 30 min of restraint stress reverses hippocampal LTP impairments and increases AMPAR GluA1 phosphorylation and surface expression. These effects persisted for more than 3 h. Moreover, we confirmed that the effects of the stress were due to glucocorticoid activation, as treatment with dexamethasone produced similar results. These findings suggest a beneficial role of acute stress on hippocampal synaptic plasticity.

Previous studies have shown that acute stress facilitates LTP by modulating glutamatergic receptors. For example, it has been shown that acute stress induced enhancement of glutamatergic transmission depends on the modifications of NMDA and AMPA receptors^25^. In mice, exposure to 30 min restraint stress shown to elevate hippocampal CA1 LTP levels through increased surface expression and GluA1 phosphorylation^26^. Furthermore, acute stress induced by immobilization and tail-shock, reported to increase the excitability of CA1 pyramidal neurons^30^, which plays an essential role in the hippocampal LTP induction. Acute stress was also shown to facilitate long-term potentiation of population spikes (PS-LTP) in the CA1 region of mouse hippocampus^31^. Similarly, acute restraint stress elevates acetylcholinesterase levels and enhances LTP in the CA1 region of the hippocampus^32^. Consistent with these observations, we also found increased surface expression of GluA1 containing AMPAR and elevated LTP in transgenic mice hippocampus which furtherly supports the LTP enhancing effect of acute stress.

A stressful brief period of swimming or immobilization elevates CaMKII levels, which are essential for AMPAR trafficking and LTP maintenance, in rat hippocampus^33,34^. We show here that acute restraint stress enhanced surface expression of GluA1 subunits of AMPARs as well as their phosphorylation. These support the phosphorylation of GluA1 subunit at S845 and their homomeric assembly in AMPARs on the membrane surface as a mechanism by which restraint stress induces LTP^26^. Notably, GluA1 phosphorylation and AMPAR surface expression are reported to be lower in mice overexpressing APP or exposed to Aβ. For instance, exposure to soluble oligomers of Aβ results in dephosphorylation and reduced surface expression of GluA1 containing AMPARs^17^. Overexpression of APP has been shown to decrease synaptic AMPARs and depress synaptic transmission^35^. Moreover, mice overexpressing APP/PS1 gene, showed to express lower levels of AMPARs^36^. Previous reports on the effect of acute stress in AD mice showed increased levels of amyloid production which results in decreased glutamatergic transmission and LTP impairment^37,38^. However, the stress paradigms used in these studies are longer, such as several hours to several days. In the present study, we show that acute stress for 30 min enhanced the phosphorylation and surface expression of AMPARs in the hippocampi of 5XFAD and Tg2576 mice for more than 3 h. The fact that total levels of these receptor subunits were unchanged in stressed mice rules out the possibility of new receptor synthesis in response to acute stress.

Corticosterone is the major glucocorticoid responsible for the effects of stress^39^. Whereas increased or sustained glucocorticoid levels impair long-term memory, acute exposure enhances memory and cognitive performance. In mice, acute stress for 15 min showed to elevate intra-hippocampal corticosterone levels up to 240 nM^40^. In addition, 30 min exposure to corticosterone or dexamethasone at 200 nM concentrations shown to enhance LTP induction, through facilitated AMPAR trafficking in rat hippocampus^26^. Similarly, a short exposure to corticosterone increases the surface mobility and synaptic content of the AMPAR GluA2 subunit in rat hippocampal neurons^41^. Accordingly, we showed that dexamethasone elevated the phosphorylation and surface expression of GluA1-containing AMPARs and enhanced LTP in the CA1 regions of 5XFAD and Tg2576 mice.

In summary, the present study demonstrated an alleviation of impaired LTP and synaptic plasticity in the hippocampal CA1 following acute stress in the AD mouse models. We show that acute restraint stress, via glucocorticoid activation, enhances LTP by facilitating AMPAR trafficking and excitatory synaptic transmission in this region. Thus, we suggest that these molecular alterations modulated by acute stress may benefit memory function and cognition in these AD mice.

## Materials and Methods

### Animals

Eleven male wild-type mice (C57BL/6 J, 7–8 weeks) and 23 Tg2576 (APP KM670/671NL, 8 months old; Taconic, Rensselaer, NY) and 24 5XFAD (APP KM670/671NL [Swedish], APP I716V [Florida], APP V717I [London], PSEN1 M146L, PSEN1 L286V, 4 months old; Jackson Laboratory, Bar Harbor, ME) male mice on a hybrid C57BL/6 J background were utilised for the experiments. Animals were housed in individually ventilated cages with free access to food and water. The animal room was controlled with a 12 h light/dark cycle (lights on at 8:00 a.m.), and the temperature was maintained at 22–30 °C.

### Restraint stress and hippocampal slice preparation

Mice were physically restrained in well-ventilated 50 ml Falcon tubes for 30 min. Control mice were housed in their usual cages under normal conditions. Animals were sacrificed immediately following restraint stress, (between 9:00 a.m. and 10:00 a.m.), and the brains were quickly removed and transferred to ice-cold artificial cerebrospinal fluid (aCSF; 124 mM NaCl, 3 mM KCl, 26 mM NaHCO_3_, 1.25 mM NaH_2_PO_4_, 2 mM CaCl_2_, 1 mM MgSO_4_ and 10 mM glucose) (Fig. 1a). For each mouse, a mid-sagittal cut was made in the brain, and one hemisphere was returned to ice-cold aCSF until required. Transverse hippocampal slices (400 μm thick) were cut using a McIlwain tissue chopper (Mickle Laboratory Engineering Co. Ltd., Guildford, UK) and allowed to stabilise in aCSF for 1 h with perfusion of 95% O_2_ and 5% CO_2_ at room temperature.

### Electrophysiology

Hippocampal slices recovered for approximately 60 min after the slice procedure to allow stable responses to be obtained. Two stimulating bipolar electrodes comprising 66 μM twisted nichrome wire were set on the Schaffer collateral pathway (for LTP input) and subiculum region (for control input). Extracellular field potentials were recorded in the CA1 region using microcapillary electrodes containing NaCl (3 M). Stimuli were delivered alternatively to the two electrodes (0.016 Hz each). After establishing a stable baseline for 30 min, LTP was evoked by two trains of tetanic stimuli (100 Hz for 1 s with a 30 s interval) and fEPSPs were recorded for at least 60 min. The slope of the evoked field potential response was measured and expressed relative to the normalised preconditioning baseline. Data were collected by a NI USB-6251 data acquisition module (National Instruments, Austin, TX), amplified by an Axopatch 200B amplifier (Axon Instruments, Foster City, CA) and captured and analysed using WinLTP software (www.winltp.com).

### Biotinylation and streptavidin pull-down assays

Surface biotinylation in acute slices was performed as described previously with some modifications^42^. Briefly, slices were washed twice in aCSF and then incubated in aCSF containing 1 mg/ml sulpho-NHS-SS-biotin for 45 min at 4 °C to label surface membrane proteins. Biotinylated tissue was then homogenised in lysis buffer containing 25 mM Tris (pH 7.6), 150 mM NaCl, 1% NP-40, 1% sodium deoxycholate, 0.1% SDS, 1 mM NaF and a cocktail of protease inhibitors (Sigma-Aldrich, St. Louis, MO). The lysate was centrifuged at 11,000 × *g* for 15 min at 4 °C to remove nuclei and cellular debris. Total protein concentration was determined with a bicinchoninic acid assay (Pierce of Thermo Fisher Scientific, Waltham, MA). A small amount of the lysate was removed for later whole-cell analysis. Subsequently, 100 μl of streptavidin beads (Thermo Fisher Scientific) was added to 600 µg of protein lysate, and the mixture was placed on a rotator at 4 °C for 2 h. Samples were then washed five times in wash buffer (25 mM Tris [pH 7.6], 150 nM NaCl, 0.5% Triton X-100), and the beads were pulled down after each wash by gentle centrifugation. Bound proteins were eluted in 2× SDS reducing buffer and gently heated at 60 °C for 30 min. The resulting supernatant was transferred to new tubes and heated at 90 °C for 5 min before gel loading.

### Immunoblotting

Hippocampal tissue lysates were prepared using radio immune precipitation buffer with protease inhibitors (Cell Biolabs, Inc., San Diego, CA). Protein concentrations were determined with a bicinchoninic acid assay. Proteins were resolved in 10–12% gels and transferred to polyvinylidene difluoride membranes (Millipore, Bedford, MA). After blocking with 1× RapidBlock solution (Amresco of VWR, Radnor, PA) for 5 min at room temperature, membranes were incubated with the following primary antibodies (Cell Signaling Technology, Danvers, MA) overnight at 4 °C: anti-pS845-GluA1 (1:1,000 dilution), anti-GluA1 (1:1,000) and anti-β-actin (1:1,000). After washing, the membranes were then incubated with horseradish peroxidase-conjugated secondary antibodies (1:5,000) in 1× RapidBlock solution for 1 h at room temperature and visualised using an enhanced chemiluminescence detection system (Millipore, Bedford, MA). The optical density of immunoreactive bands was measured using ImageJ software (National Institutes of Health, Bethesda, MD). The results were normalised to the quantity of β-actin in each sample lane.

### Statistical analysis

All statistical graphs represent means ± the standard errors of the means (SEMs). Data were analysed from one slice per mouse (*n* = number of slices = number of mice). Relative percentages of LTP and protein expressions were presented as tables at the end of the manuscript (Tables 1–3). Statistical analysis was performed either by a two-tailed unpaired Student’s *t* test to compare two groups or a one-way ANOVA with post-hoc test when ≥ 3 groups were compared using SPSS 23.0 software. A *P* value of <0.05 was considered statistically significant.

**Table 2:** Time dependent effect of acute stress on LTP, and phosphorylation of AMPA - GluA1 levels.

	fEPSP slope				pS845-GluA1			
	control	post stress			control	post stress		
		1 h	3 h	5 h		1 h	3 h	5 h
5XFAD	102 ± 7%	170 ± 8%	153 ± 7%	108 ± 2%	100%	250 ± 8%	219 ± 1%	106 ± 1%
Tg2576	118 ± 5%	176 ± 7%	156 ± 5%	120 ± 5%	100%	224 ± 9%	194 ± 1%	110 ± 2%

**Table 3:** Effect of dexamethasone on LTP, Surface expression and phosphorylation of AMPA - GluA1.

	fEPSP slope		sGluA1		pS845-GluA1	
	C	Dex	C	Dex	C	Dex
5XFAD	102 ± 7%	161 ± 5%	100%	217 ± 1%	100%	232 ± 8%
Tg2576	118 ± 5%	171 ± 4%	100%	250 ± 8%	100%	214 ± 8%

### Study approval

All experiments and methods were performed in accordance with the relevant guidelines and regulations. Experiments involving animals followed approved protocols from the Institutional Animal Care and Use Committee of Chonnam National University.

## Supplementary information

Supplementary information

## Data Availability

The data sets generated and/or analysed during the present study are available from the corresponding author on reasonable request.
