# Supplementary material for: Acute restraint stress reverses impaired LTP in the hippocampal CA1 region in mouse models of Alzheimer’s disease
Source: Sci Rep. 2019 Jul 29;9:10955. doi: 10.1038/s41598-019-47452-6 (PMC6662902; doi:10.1038/s41598-019-47452-6)

**Supplementary information**

**Acute restraint stress reverses impaired LTP in the hippocampal CA1 region in mouse models of Alzheimer’s disease**

Ming Wang^1,2#^, Vijay Sankar Ramasamy^1,2#^, Manikandan Samidurai^1,2^ and Jihoon Jo^1,2,3^*

^1^NeuroMedical Convergence Lab, Biomedical Research Institute, Chonnam National University Hospital, Jebong-ro, Gwangju 501-757, Republic of Korea.

^2^Department of Biomedical Sciences, BK21 PLUS Center for Creative Biomedical Scientists at Chonnam National University, Research Institute of Medical Sciences, Chonnam National University Medical School, Gwangju 501-757, South Korea.

^3^Department of Neurology, Chonnam National University Medical School, Gwangju 501-757, Republic of Korea.

**Supplementary Figure 1. Acute stress enhances LTP in WT mice**

Tetanic stimulation (two trains of 100 Hz, 100 pulses) was delivered to induce LTP in CA1 neurons after 30 min of baseline recordings. Exposure to 30 min of restraint stress enhanced LTP. Error bars represent the SEMs (*n* = 5 control, 6 stressed).

**Supplementary Figure 2. Acute stress enhances GluA1 phosphorylation and surface expression in WT mice**

(**a**) Representative immunoblots and densitometry analysis showing GluA1 surface expression in hippocampi of WT mice (*n* = 3/group). (**b**) Representative immunoblots and densitometry analysis showing the amount of pS845-GluA1 and total GluA1 in hippocampi from WT mice (*n* = 4/group). C, unstressed control; S, stressed. Error bars indicate SEMs; **P* < 0.05; ***P* < 0.01. Full length blots are presented in Supplementary Figure 6.

**Supplementary Figure 3.**

Full-length western blots of surface, phospho- and total GluA1 expression in acute stress exposed 5XFAD and Tg2576 mice hippocampal preparation. Black squares show the cropped bands.

**Supplementary Figure 4.**

Full-length western blots of phospho- and total GluA1 expression at different time points in acute stress exposed 5XFAD and Tg2576 mice hippocampal preparation. Black squares show the cropped bands.

**Supplementary Figure 5.**

Full-length western blots of surface, phospho- and total GluA1 expression in dexamethasone exposed 5XFAD and Tg2576 mice hippocampal preparation. Black squares show the cropped bands.

**Supplementary Figure 6.**

Full-length western blots of surface, phospho- and total GluA1 expression in acute stress exposed wild type mice hippocampal preparation. Black squares show the cropped bands.

**
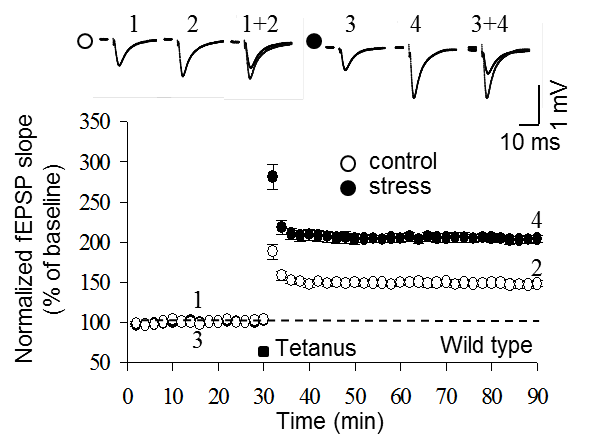
Supplementary Fig. 1.**

**
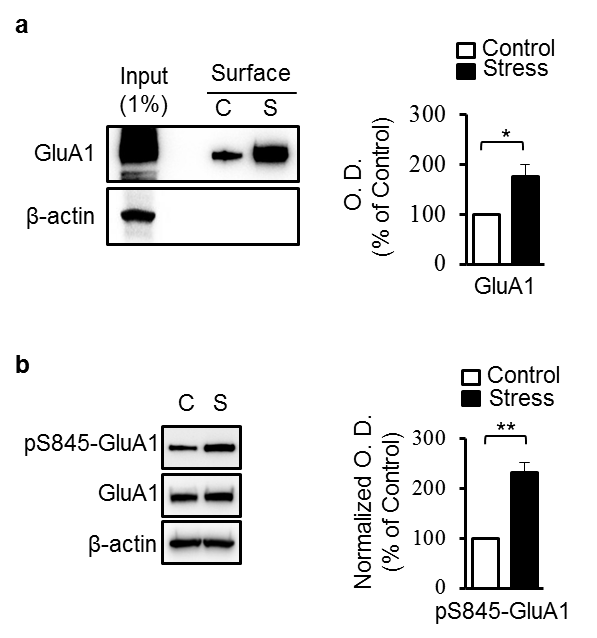
Supplementary Fig. 2.**

**Supplementary Figure. 3.**


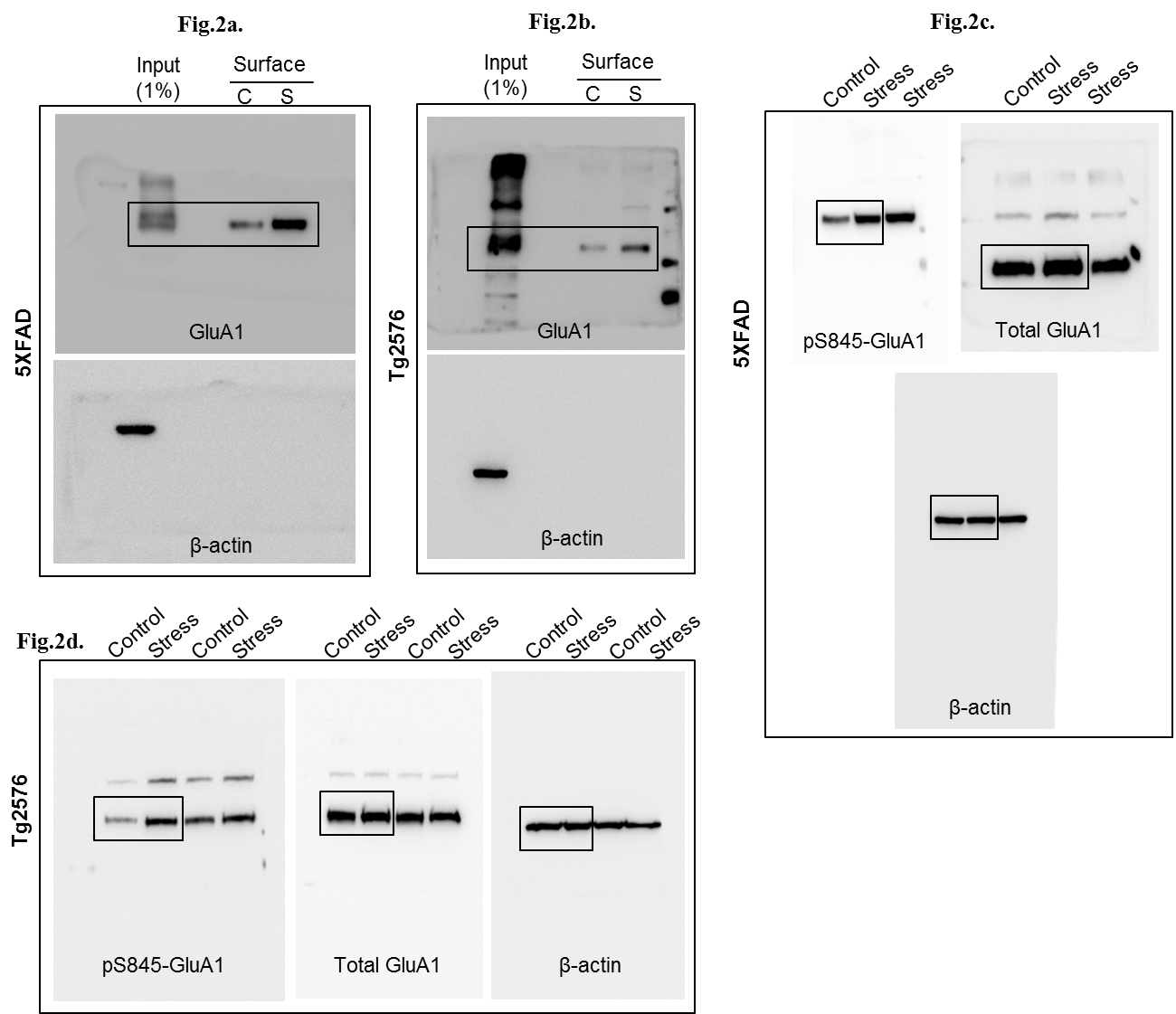


**Supplementary Figure. 4.**


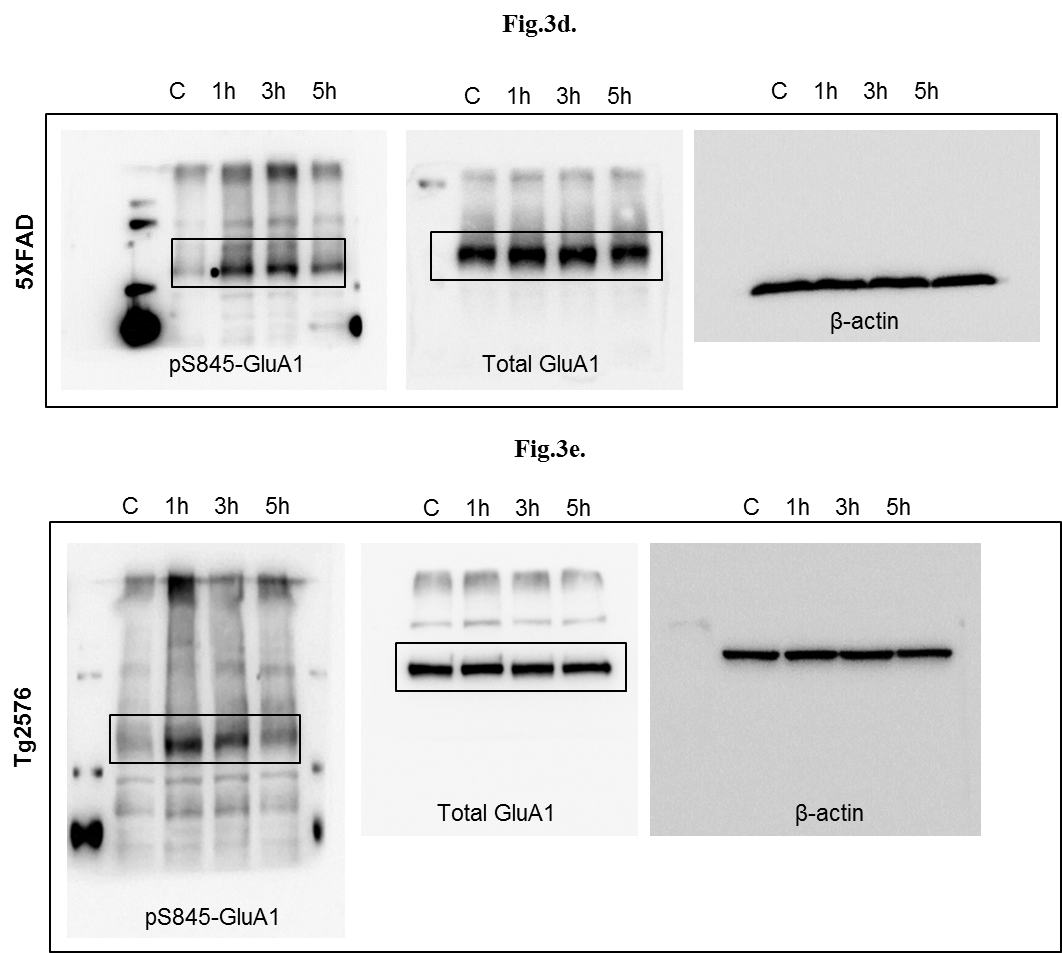


**Supplementary Figure. 5.**


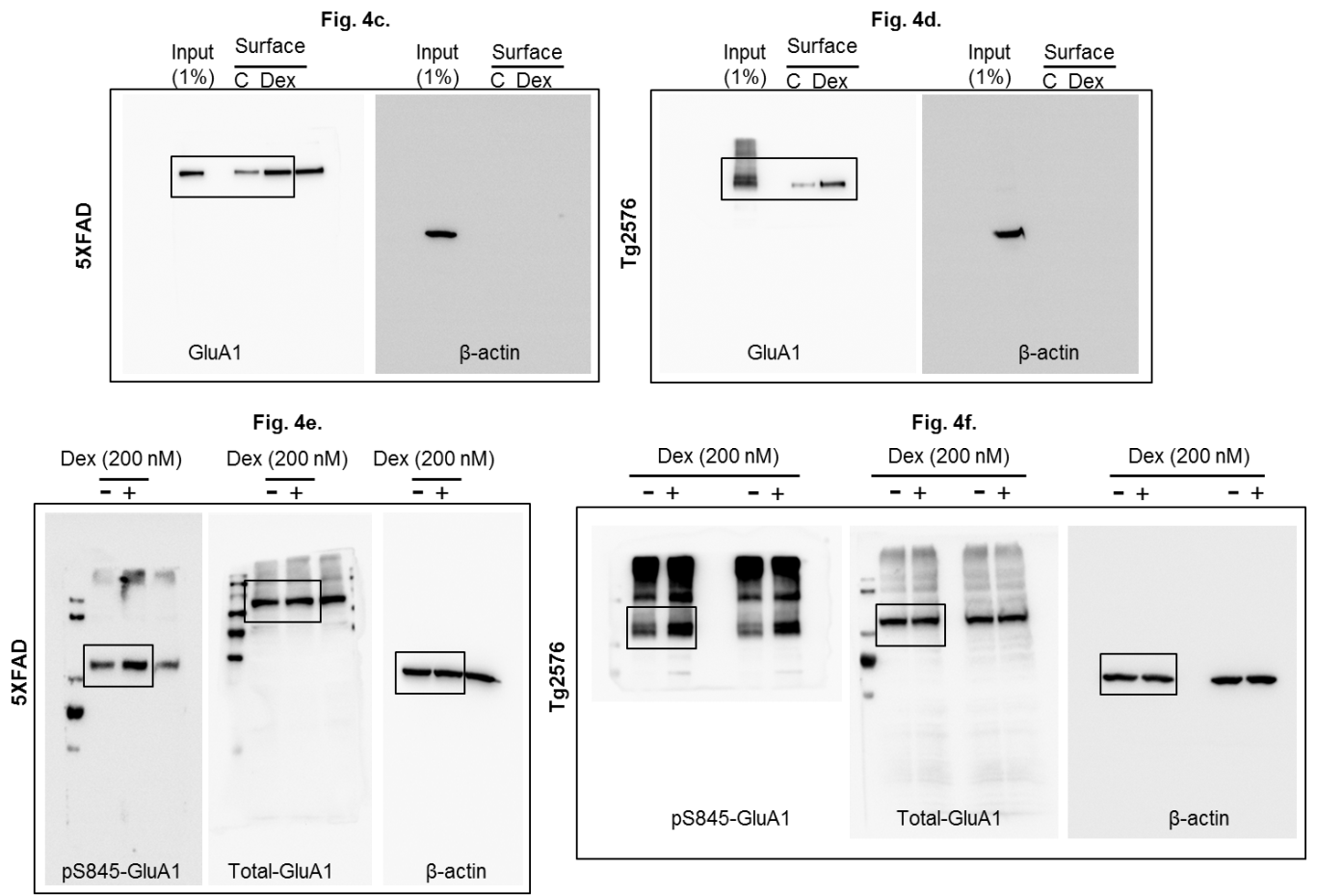


**Supplementary Figure. 6.**


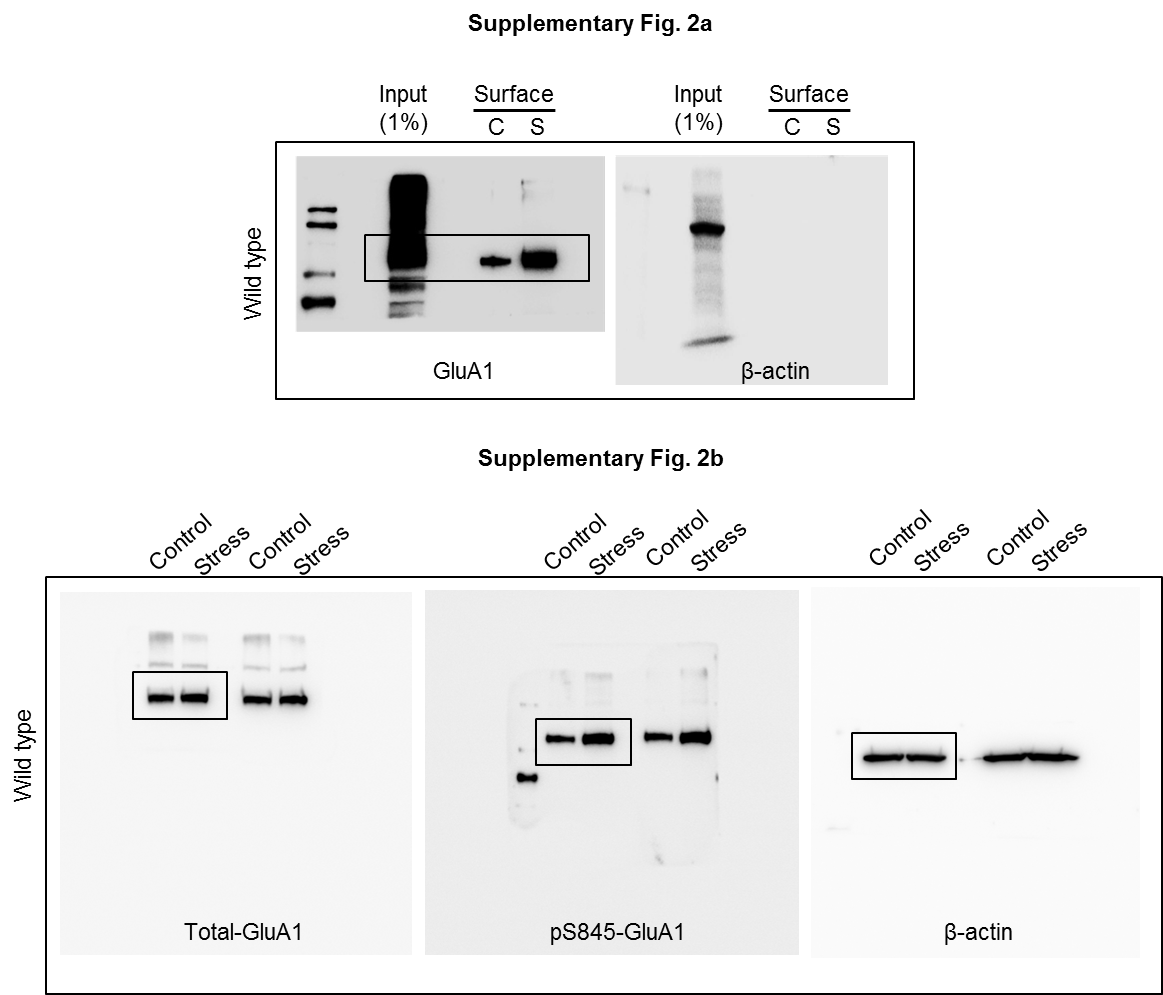

Supplement: Supplementary file 1 — Supplementary information [file 41598_2019_47452_MOESM1_ESM.docx]
